# Do foreign bodies migrate through the body towards the heart?

**DOI:** 10.1016/S1808-8694(15)30778-3

**Published:** 2015-10-19

**Authors:** Rui Celso Martins Mamede, Fabiana do Amaral, Daniel Garcia Raimundo, Luiz Carlos Conti de Freitas, Hilton M.A. Ricz, Francisco V. Mello-Filho

**Affiliations:** 1 Associate Professor–Department of Ophthalmology, Otorhinolaryngology and Head and Neck. Head of the Head and Neck service–HCFMRP-USP; 2 MD. ENT resident; 3 MD. ENT resident; 4 MD; 5 PhD. Professor; 6 PhD. Professor

**Keywords:** esophageal diseases, esophagus, pharynx

## Abstract

Fixation of foreign bodies (FB), in the mucosa, can favor its migration, giving origin to the popular saying: “FB walk to the heart”.

**Aim:**

describe the mechanisms involved in FB migration and how to diagnose them.

**Methodology:**

From a sample of 3,000 foreign bodies, during 40 years, we analyzed four which had extra-lumen migration. We analyzed clinical, radiologic, endoscopic and ultrasound data collected at the medical documentation service.

**Results:**

three clinical histories are presented, describing two fish bones and one piece of fish cartilage. FB shifting was analyzed in all of them. Migration started in the esophagus in two, one going to the aorta and the other to the neck area. In the other two, migration started in the pharynx, and the FB moved towards the prevertebral fascia and the other externalized in the submandibular region. The mechanisms and the risks posed to the patient, by FB migration, and the way to diagnose them are hereby discussed.

**Conclusions:**

the study allows us to determine that FB can move through the body but not towards the heart. The study also serves as a warning sign: in cases of prolonged histories of FB ingestion, imaging studies are mandatory before endoscopic examination.

## INTRODUCTION

It is known that foreign bodies (FBs) aspirated to the airways or which get stuck during swallowing in the digestive pathway are nor rare. It is believed that they happen in a frequence of 0.5/100,000 people, in other words, about 1,000 cases per year in Brazil. Those aspirated to the airways have to be removed by bronchoscopy, because only 2% are spontaneously eliminated. Most of the ones which are swallowed end up being eliminated together with feces; nonetheless, the larger or pointed ones can get stuck in the pharynx or esophagus, causing symptoms which require their removal by esophagoscopy. In some cases, after getting fixed to the digestive pathway, they can cause perforation, abscess or even damage to the great vessels, characterizing a FB shifting through the neighboring tissue. From this observation resulted the popular saying that “FBs move inside the body towards the heart”.

Given that, the goal of the present investigation is to describe the mechanisms associated with FBs which moved around the body and the means to diagnose them.

## METHODOLOGY

Having a total of about 3,000 cases of foreign bodies (FB) treated in the University Hospital, a tertiary facility, in a period of 40 years, we selected those that presented signs of extraluminal shifting. Those were four patients who had an FB attached to their digestive tracts. The patients were identified based on the information collected from the medical documentation service. We collected data associated with signs and symptoms, results of endoscopic and radiographic exams, CT scan and ultrasound (US). FB migration was proven by means of radiographic signs (presence of air, secretion or visualization of the FB in the neighboring tissues) and/or endoscopic signs (presence of purulent secretion or absence of all or part of the FB in the intraluminal compartment or even organ perforation).

## RESULTS

Our sample was made up of 3 men and one woman, with ages varying between 38 and 55 years, one in the 70's, another one in the 80's and the last two in 2005.

The first was male, 55 years of age, with a history of having swallowed a fish bone seven days prior to his visit. He complained of retrosternal pain during swallowing, even when he swallowed saliva. A conventional RX image did not show anything wrong. He was submitted to esophagoscopy under general anesthesia, and then the physician saw the fish bone in the middle third of the esophagus. The FB removal was followed by intensive bleeding, and the patient died of it subsequently.

The second patient, male, diabetic, 53 years old, complaining of a fish bone stuck in his pharynx, which he had tried to remove with his fingers. The lateral view radiography showed the FB located behind the larynx, which was not found during the endoscopic exam under general anesthesia. However it was seen closer to the cervical spine when a contrasted x-ray was done together with the endoscopy. The patient ended up having a retropharyngeal abscess with glucose decompensation and was admitted to the ICU, where he stayed for 6 months. The abscess drainage pushed the FB to the digestive tract, and it was expelled.

**Figure 1:**
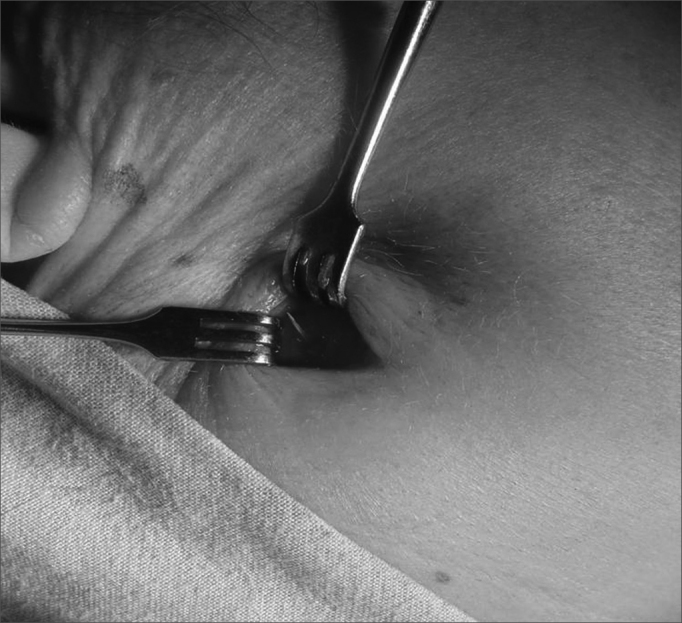
Linear calcic image of the cricoid, medially and below the carotid, with a small quantity of air around it.

The third patient was also a diabetic male, 38 years old, who had swallowed a fish bone 14 days prior to his visit and complained of dysphagia and odynophagia. He reported having been submitted to endoscopy, and the physician only found purulent secretion at the entrance of the esophagus and the CT scan carried out at that service showed retropharyngeal air from the skull base all the way to the sternum furcula (Figure 1). He was admitted to the ICU and received antibiotic treatment before coming to us. In the hospital, both the esophagoscopy and the image study confirmed the patient's report; however, it was possible to see an image considered a fish cartilage outside the esophagus, near the larynx. Through a skin incision on the anterior border of the left sternocleidomastoid muscle, the FB was removed, together with serum and bloody secretion, very near the carotid. During the procedure we did not see any perforation on the esophageal wall.

**Figure 2:**
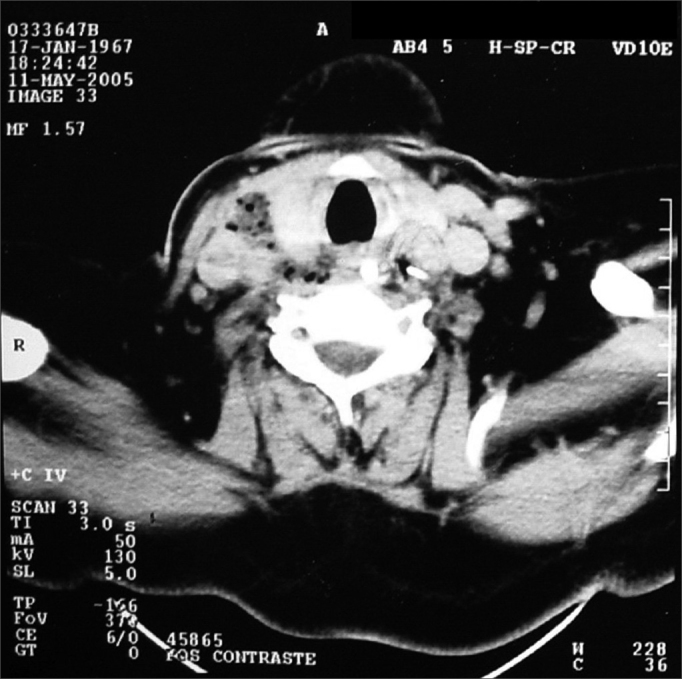
FB coming out in the submandibular region.

The fourth patient was female, with 51 years of age, who came 14 days after having swallowed a fish bone and who was treated with antibiotics in another hospital. She complained of submandibular pain, where there was a bulged area of 3cm in diameter with signs of inflammation (redness, heat and pain). CT scan reveals a FB. By means of a small skin incision the fish bone was exposed and removed (Figure 2). The patient was cured.

## DISCUSSION

Since the esophagus is the narrowest organ of the digestive tract, the FBs which manage to pass through the gastroesophageal junction, usually move forward freely and become part of the feces. The FBs which are large in comparison to the esophagus diameter get stuck at any point of the upper portion of the digestive tract; e.g. coins and rings get stuck to the esophagus, while dental prosthesis get stuck in the retrocricoid region. Among those which get stuck to the esophagus, most remain stuck below the Upper Esophageal sphincter (UES) and not above it. This is not true for the gastric-esophageal transition zone, where the FBs get stuck above the LES. This happens because when the laryngeal phase of swallowing starts, the upper esophageal sphincter opens with the laryngeal movement upwards, thus, when the FB reaches the IES, it does not prevent its passage. We add to this the fact that the FBs are pushed together with the food by powerful movements of the tongue and pharyngeal constricting muscles.

Pointed objects can get stuck on the pharynx (cases 2 and 4) and in the esophagus (cases 1 and 3), and more rarely in other sites of the digestive tract. In the pharynx, it usually happens on the vallecula or the lymphoid tissue, especially in the hypertrophied tonsil or tongue base^1^^,^^2^. In the esophagus, it usually gets stuck in the aortic-bronchial notch of its middle third (case 1). Once settled, FBs cause symptoms of irradiated pain, dysphagia and odynophagia which, because of its intensity, cause the patient to look for medical treatment. Flexible or rigid esophagoscopy shows these FBs in 94% of the times and allows for its removal, curing the patient (Shinhar et al.^3^).

It is a fact that some FBs end up perforating the pharynx or esophagus, causing a very dangerous clinical situation because of the incidence of morbidity and mortality (Silva and Ahluwalia^4^). Before the perforation, there is FB fixation on the organ's walls, causing periesophagitis or peripharyngitis, which can evolve to become an abscess. Perforation is followed by: chest pain, fever, subcutaneous emphysema, dyspnea and dysphagia, and pain and fever are the most frequent, since they are present in 90% of the cases^4^, ^5^, ^6^, ^7^. These symptoms do not help to achieve a clear diagnosis, because the symptoms overlap those of other disorders of this anatomical region, or even those of intraluminal foreign bodies (with or without abscess). The long duration of symptoms was, in the cases hereby presented, the only hint that it was not an intraluminal FB, since it was the radiographic and endoscopic exams that allowed for a definitive diagnosis of extraluminal FBs. Symptoms were present in all our cases, however there can be asymptomatic esophagus perforation, and there are four cases like this reported in English in the literature. The authors were not convincing in their explanation for this lack of symptoms^4^^,^^8^.

The perforation of the digestive tract is common after instrumentation (endoscopy, intubation, dilations) and surgery, and they are both responsible for up to 80% of the cases^4^. Silvis et al.^9^ and Spechler et al.^10^ detected that the risk of perforation during endoscopic exam is of 0.03%; of dilation it is 0.1–0.35% and achalasia dilation with balloon is between 2–6%. Spontaneous rupture (Baerhaave Syndrome); trapped hiatal hernia perforation or ingestion of caustic products can also cause such symptoms. Among the cases of perforation, the perforation caused by the presence of a FB is rare, varying between 7 and 17%^5^^,^^7^^,^^11^, and it is more frequent among pointed FBs. In our study, in a tertiary hospital, we had 4 cases among some 3,000 of FB seen in 40 years of activities and they were all pointed FBs. Nonetheless, we find in the literature cases of non-pointed FBs causing perforation, such as coins^12^ and nuts skins^13^ which invade the thyroid and the mediastinum, respectively.

According to Eroglu et al.^5^, the chest has 2/3 of the esophageal perforations, while the neck has the other third. The poor blood supply to the esophagus middle third could explain such phenomenon. However, the perforation caused by FB impaction happens more frequently in the hypopharynx and the cervical esophagus, because it has the influence of tongue base and pharyngeal muscles which are stronger muscles4. Of the four cases hereby reported, only one happened in the thoracic portion of the esophagus.

Usually, the treatment of the infection after FB diagnosis and removal can heal the patients with perforation, with or without sequelae. However, some may present with atypical evolution, as the ones presented in this study, that is, they cross the organ's wall and migrate through the extraluminal tissue^4^^,^^12^^,^^14^^,^^15^. During such migration, they may rupture large vessels such as the carotid, aorta or damage neighboring organs, such as the thyroid gland.

Esophageal perforation can be followed by pleural effusion; pneumomediastinum; hydropneumothorax; air in the subdiaphragmatic space; aspiration pneumonia (in the presence of tracheoesophageal fistula); arterial bleeding (when there is aorta injury^5^)(aortic-esophageal fistula) or carotid 16 (carotid-pharyngeal fistula), or cardiac tamponage^17^ (should the pericardium be perforated) and mediastinitis^18^. Pharyngeal perforation is followed by neck emphysema and carotid-pharyngeal fistula (should there be carotid injury). According to Bladergroen et al.^7^, mortality in these cases is of 21%.

Radiographs can detect perforations in 90% of the cases when there are signs of complications. Contrast overflowing is not always present, and it can fail the diagnosis in 80% of the cases when the perforation is in the chest region and in 50% of the times when it is in the neck region19. CT scan can be useful, especially when the X-ray is negative^20^. At the time, there was no CT scan for cases 1 and 2. In cases 3 and 4, the CT scan showed the FB, but only when performed in our hospital. This shows that besides the CT scanner, there is the need of skilled specialists to interpret the signs provided by the images.

The sight of the perforation during the endoscopic exam is highly sensitive, however air inflation can cause intramural esophageal dissection, worsening the patient's condition^21^. We stress that in some cases, after FB migration, the esophageal wall is scarred and the FB passage hole closes up without a trace (cases 2, 3 and 4). For Silva and Ahluwalia^4^ perforation treatment is not surgically treated if it is very small and located in the neck region.

Pointed FBs migrate through the tissues of the neck and chest, because once fixed to the walls, they are pushed by the food ingested (case 1) or by careless maneuvers used to try to remove it (case 2), that being by a finger, NGT, balloons, etc. Thus, they end up crossing the organ's wall. In this process, there is an important participation of the body's skeletal muscles, which happens when one moves one's head, turning it sideways or flexing or extending it. Even physiologic actions such as swallowing and coughing or those that count on the participation of distant muscle groups, such as seating, squatting, defecating, etc., end up increasing the pressure on the neck and chest regions, favoring FB shifting. Thus pushed, the FBs penetrate deep in the organ, gaining the extraluminal space (cases 3 and 4). After crossing the organ's wall, the hole through which it passed suffers a healing process and closes up. This seems to explain case 3 of the present investigation.

Of the cases presented in this study, only one patient died; however, the other 3 ran a serious risk of death by infection or by the possibility of the FB perforating the carotid (case 3). Therefore, we suggest that in all cases of long lasting FBs (more than 3 days), the radiographic study (including CT scan) should be routine, before endoscopic examination, in order to detect signs of perforation or FB migration. Should this be the case, these exams help make a more in-depth analysis of how to best approach these cases. If the CT scan had been carried out (they did not have it at the time), it could have indicated thoracotomy as the best approach to remove the FB in case 1.

## CONCLUSIONS

The authors believe that FBs can jeopardize the life of their bearers and they do migrate through the body, however not towards the heart. We stress that in cases of long standing FBs, image studies are Paramount before proceeding with endoscopic exams.
